# Effect of decoupling hydraulic and solid retention times on carbohydrate-rich residue valorization into carboxylic acids

**DOI:** 10.1038/s41598-023-48097-2

**Published:** 2023-11-23

**Authors:** Adrián Lago, Silvia Greses, Kaoutar Aboudi, Inés Moreno, Cristina González-Fernández

**Affiliations:** 1 Biotechnological Processes Unit, IMDEA Energy, Avda. Ramón de la Sagra 3, 28935 Móstoles, Madrid Spain; 2 Thermochemical Processes Unit, IMDEA Energy, Avda. Ramón de la Sagra 3, 28935 Móstoles, Madrid Spain; 3 Department of Chemical Engineering and Food Technology, Faculty of Sciences (Wine and Agri-Food Research Institute-IVAGRO and International Campus of Excellence-ceiA3), University of Cádiz, Republic Saharawi Avenue, P.O. Box No. 40, 11510 Puerto Real, Cádiz Spain; 4 Chemical and Environmental Engineering Group, ESCET, Rey Juan Carlos University, 28933 Móstoles, Madrid Spain; 5 Department of Chemical Engineering and Environmental Technology, School of Industrial Engineering, University of Valladolid, Dr. Mergelina, S/N, 47011 Valladolid, Spain; 6 Institute of Sustainable Processes, Dr. Mergelina, S/N, 47011 Valladolid, Spain

**Keywords:** Biotechnology, Environmental sciences, Engineering

## Abstract

This research assessed the effect of decoupling hydraulic retention time (HRT) and solid retention time (SRT) on the production of volatile fatty acids (VFAs) via anaerobic fermentation of beet molasses. The performance of a continuous stirred tank reactor (CSTR, STR = HTR = 30 days) and two anaerobic sequencing batch reactors (AnSBR) with decoupled STR (30 days) and HRT (20 and 10 days) was compared. Previously, a temperature study in batch reactors (25, 35, and 55 °C) revealed 25 °C as the optimal temperature to maximize the VFAs yield and the long-chain VFAs (> C_4_) production, being selected for the continuous reactors operation. An HRT of 20 days in AnSBR led to an enhancement in bioconversion efficiency into VFAs (55.5% chemical oxygen demand basis) compared to the CSTR (34.9%). In contrast, the CSTR allowed the production of valuable caproic acid (25.4% vs 4.1% w/w of total VFAs in AnSBR). Decreasing further the HRT to 10 days in AnSBR was detrimental in terms of bioconversion efficiency (21.7%) due to primary intermediates (lactate) accumulation. By decoupling HRT and SRT, VFAs were maximized, revealing HRT as an effective tool to drive specific conversion routes (butyrate- or lactate-fermentation).

## Introduction

Volatile fatty acids (VFAs) are a group of short-chain carboxylic acids including acetic acid, propionic acid, butyric acid, isobutyric acid, valeric acid, isovaleric acid, and caproic acid, which are considered key building blocks for the chemical industry. These carboxylates are precursors of a wide range of valuable products such as cosmetics, food additives, pharmaceuticals, rubbers, lubricants, biofuels, and biopolymers^1–3^. Nowadays, VFAs are mainly produced via non-sustainable petrochemical processes with concomitant greenhouse gas (GHG) emissions^4–6^. The increasing petroleum price and the objectives marked by the European Commission to reduce GHG emissions by at least 55% by 2030^7^ raised the need to develop alternative technologies to produce this type of chemicals via more sustainable pathways. In this context, biological processes have recently gained much attention to obtain VFAs from renewable sources, such as organic wastes.

Anaerobic digestion (AD) is one of the most utilized bioprocesses for the valorisation of organic-rich residues to produce bioenergy in the form of biogas^8,9^ or biohydrogen^10,11^. Yet, the required paradigm change related to the production of VFAs from renewable sources has drawn the attention of this technology for carboxylates production^12,13^. AD consists of four main steps: hydrolysis, acidogenesis, acetogenesis, and methanogenesis. Conventionally, AD treatment has been oriented to biogas production (methane and carbon dioxide)^12^. However, this bioprocess can be adjusted to hamper the methanogenesis step, leading to the accumulation of VFAs and preventing biogas production. In order to steer the process toward VFAs accumulation, recent studies investigated the anaerobic fermentation (AF)^14–17^, namely constituted by the hydrolysis and acidogenesis steps. The manipulation of operating parameters set in the reactors has been elucidated as efficient strategies to promote only the fermentative stages of AD. In this regard, slightly acidic pH (5–6)^8,12^, mild temperatures (20–35 °C), high organic loading rates (OLRs) (3–20 g_COD_ L^−1^ day^−1^) ^18,19^ and low hydraulic retention times (HRTs) (< 15 days) have been reported as inhibitors of the methanogenic activity, boosting the accumulation of VFAs^20^ in continuous stirred tank reactors (CSTRs). Nevertheless, the effect of solid retention time (SRT) on AF performance has been poorly studied.

CSTR is the most widely used configuration in which, the HRT and the SRT are equal due to the absence of a biomass recirculation system. Whereas biogas production in AD requires long HRTs (20–30 days) to promote methanogenic archaea growth, short HRTs (< 15 days) are used when targeting carboxylates production. This strategy is based on the fact the archaea growth rate (VFAs consumers) is slower than that of anaerobic bacteria^20,21^ (i.e. *Methanosarcina* and *Methanosaeta* archaea show growth rates of 0.43 day^−1^ and 0.12 day^−1^ respectively, while *Acetobacterium* bacteria has a growth rate of 1.104 d^−1^)^22^. Nevertheless, short HRTs might also provoke the washout of bacteria involved in the fermentative stage, reducing the AF efficiency. To overcome the key bacteria washout without compromising the AF cost-effectiveness, HRT and SRT can be decoupled by using alternative reactors configuration such as up-flow anaerobic sludge blanket reactors (UASB), anaerobic sequencing batch reactors (AnSBR) or packed bed biofilm reactors^1,23,24^. AnSBR represents one of the most economical reactor configurations to decouple HRT and SRT since pumping and extra elements (i.e. membranes, setting tank) are not required^25,26^. The fill-react-draw nature of the AnSBR allows treating a high volumetric loading rate while enabling significant microbial biomass retention^27^. In this way, carboxylate productivity can be enhanced by feeding high influent flow rates (lowering HRT) while keeping the microbial biomass long enough (long SRT) to attain high conversion of organic matter into VFAs. It is important to highlight that temperature and pH might also play an important role in avoiding methanogenic activity when long SRTs are implemented^28^. As methanogens are the most sensitive microorganisms in the AD^29^, process temperatures lower than 35 °C (i.e. 25 °C) significantly limit the methanogenic growth rate^12^ and reduce VFAs consumption to produce methane. Similarly, pHs below neutrality also promote VFAs accumulation due to the high sensitivity of methanogens upon pH changes^30^. Nevertheless, optimal conditions highly depend on the feedstock composition.

Among the wide variety of organic wastes used as feedstock in AF, such as cheese whey^23^, agroindustrial wastes^8^, food wastes, industrial wastewater^31,32^ or grease^33^, molasses represents a promising substrate for carboxylates production. Molasses is a byproduct of the sugar industry which consisted of a thick syrup containing non-extractable sugars from sugar cane or sugar beet. Molasses has been normally used as a raw material in fermentation industries to produce yeast, vitamins, amino acids, citric and lactic acid^34^, and especially, for bioethanol production (70% of total molasses production in the EU)^35^. Its composition, rich in carbohydrates (up to 50%^36^), mainly sucrose^37^, made molasses an easily degradable substrate suitable for maximizing VFAs production via AF processes.

Thus, this research aimed at first elucidating the optimal process temperature to valorize a carbohydrate-rich residue into VFAs. Thereafter, the effect of decoupling HRT to SRT on the VFAs production yields and distribution profile was assessed by comparing the operation in a CSTR and an AnSBR.

## Materials and methods

### Residue used as feedstock and inoculum

Beet molasses, kindly supplied by Compañía de Melazas S.A (Madrid, Spain), was selected as a feedstock due to their high content of carbohydrate and readily biodegradable organic matter. In Table 1, the characterization of beet molasses was shown in terms of total and soluble chemical oxygen demand (Total COD (TCOD) and soluble COD (SCOD), respectively), total and volatile solids (TS and VS, respectively), pH, carbohydrate, protein, lipid and ash contents.

**Table 1:** Chemical characterization of the beet molasses used as feedstock (mean ± SD) (n = 3).

	Beet molasses
TCOD (g L^−1^)	1028 ± 18
SCOD (%)	98.6 ± 0.7
TS (g L^−1^)	1067 ± 1
VS (%)ª	85.7 ± 0.5
pH	7.0 ± 0.1
Carbohydrates (wt%)^a^	56.6 ± 0.8
Proteins (wt%)^a^	12.5 ± 1.2
Lipids (wt%)^a^	16.7 ± 0.7
Ash (wt%)^a^	14.2 ± 0.5

^a^Calculated in a dry matter basis.

The anaerobic sludge used as inoculum in batch tests and continuous reactors was collected from a conventional AD process located in a municipal wastewater treatment plant (El Soto-Móstoles, Spain). In this plant, the AD was performed at 35 °C and the inoculum presented the following composition: 11.7 ± 0.3 g TS L^−1^, 8.2 ± 0.3 g VS L^−1^, 0.8 ± 0.1 g N-NH_4_^+^ L^−1^ and pH 7.5 ± 0.1.

### Biochemical carboxylate potential (BCP) tests

In order to identify the optimal temperature to maximize the conversion of beet molasses into VFAs, the residue was evaluated by means of BCP tests according to the procedure described by Magdalena and González-Fernández^38^. These tests were performed in 120 mL batch reactors with a working volume of 70 mL and a substrate-to-inoculum ratio of 3 g COD_substrate_/g VS_inoculum_. This ratio was selected according to previous studies in which this value provoked the AD imbalance required for methanogenic activity inhibition^39^. In parallel, blank tests using similar conditions but adding water instead of the substrate were also carried out to evaluate the endogenous VFAs production of the sludge. pH was initially adjusted at a neutral value (pH 7.0–7.3) to ensure a proper performance of the process^40^ using a NaOH solution (5 M). To evaluate the effect of the temperature, BCPs were performed at 25, 35 and 55 °C in incubators (AG CH-4103 Bottmingen, Infors). VFAs were analysed twice a week by extracting 0.5 mL of sample from the batch reactors and filtered through 0.2 µm filter. The gas production (hydrogen, carbon dioxide, and methane) was calculated by measuring the pressure in the reactors´ headspace and the composition was periodically analysed. All the experiments, including blank tests, were performed in triplicate to ensure process replicability. The experiments were run for at least 15 days until the VFAs and gas production showed stability.

### Anaerobic reactors description and operation

Once the optimal temperature was selected based on the BCPs results, the AF of beet molasses was assessed in continuous reactors under two different configurations: CSTR and AnSBR.

**Figure 1:**
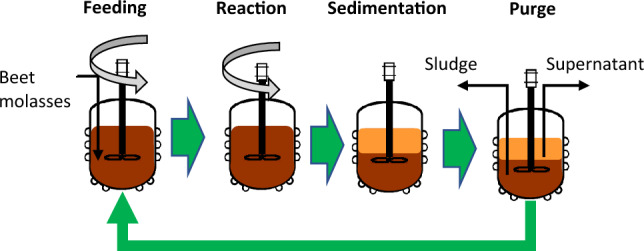
Schematic representation of the AnSBR operation cycle stages.

The CSTR consisted of a reactor of 1 L of working volume (0.5 L headspace), inoculated with the same AD inoculum as in the BCPs test. This reactor was operated at an HRT of 30 days (HRT = SRT), and the pH was adjusted between 5.5 and 6 using aqueous solutions of NaOH (5 M) or H_2_SO_4_ (5 M) when necessary. According to previous studies, these conditions ensure the inhibition of methanogenic archaea and favour the accumulation of VFAs^8,12^. The reactor was homogenized by using a magnetic stirrer. The 1.25 L-AnSBR (0.25 L headspace) was inoculated with the inoculum described above but its operation consisted of a cycle of 24 h divided into four stages (Fig. 1): feeding (15 min), reaction (600 min), sedimentation (840 min) and purge (15 min). The selected sedimentation stage period was longer in comparison to the times employed for traditional AnSBR operation of conventional AD^23,41^ due to the difficulties of this sludge to settle down and form two different phases, the supernatant and the sediment. Despite that, this inoculum was selected based on the high microbial biodiversity^42^, which provided a wide range of metabolic pathways that ensure process flexibility under different conditions. Aiming at finding the optimum trade-off between biological, chemical and physical characteristics, the use of conventional anaerobic sludge was envisaged as the most suitable strategy. In this way, after the settling period, the separation of phases allowed the decoupling of SRT and HRT. The effect of the HRT in the AnSBR was evaluated at 10 and 20 days, maintaining the SRT at 30 days (same value implemented for the CSTR). Similarly, the pH was adjusted in a range of 5.5–6.0 with NaOH (5 M) or H_2_SO_4_ (5 M) solutions when necessary. To ensure that the stationary phase has been reached, all the reactors were run for a minimum of three SRT and stopped when chemical parameters were constant. The effluents of the reactors were analysed twice a week in terms of TCOD, SCOD, TS, VS, pH, and VFAs content to monitor the AF process. Similarly to the BCP tests, biogas production and composition were also measured periodically.

### Analytical methods

pH was daily measured using a pHmeter (Hach-Lange SENSION + pH31). TCOD, SCOD, TS, VS and ash content in the feedstock and reactors were quantified according to Standard Methods^43^. The content of carbohydrates in beet molasses was measured by the phenol-sulfuric method^44^. Protein content was also analysed by determining the total organic nitrogen content by Kjeldahl method^45^ and applying a conversion factor of 6.25^46^. Lipid content was calculated by subtracting carbohydrates, proteins and ash from the total dry matter. VFAs, lactic acid and ethanol concentrations were measured by liquid chromatography using an Agilent 1260 HPLC equipped with a refractive index detector, a pre-column (Cation H Refill Cartridge Microguard column, Biorad) and an ion exclusion column (Aminex HPX-87H 300 × 7.8 mm I.D., Biorad) using the conditions described by Llamas et al.^47^. Biogas composition (hydrogen, carbon dioxide and methane) was determined by gas chromatography coupled with a thermal conductivity detector (Clarus 580 GC, PerkinElmer), equipped with an HSN6-60/80 Sulfinert P packed column (7′ 1/8ʺ O.D.) and a MS13X4-09SF2 40/60P packed column (9′ 1/8ʺ O.D.) (PerkinElmer), using the same conditions as Llamas et al.^48^.

Once the AF reached the steady state (showing stability in terms of TCOD, SCOD, VFAs, TS and VS in the effluents), the process efficiency was evaluated by calculating the bioconversion and COD_acidified_ according to the Eqs. (1) ^12^ and (2)^30^:

1 $$\% \text{Bioconversion = }\frac{{\text{COD-VFAs}}_{\text{effluent}}}{{\text{TCOD}}_{\text{influent}}} \, \times \text{ 100}$$

2 $$\% {\text{COD}}_{\text{acidified}}\text{ } = \frac{{\text{COD-VFAs}}_{\text{effluent}}}{{\text{SCOD}}_{\text{effluent}}} \, \times {100}$$

where COD-VFAs_effluent_ referred to the summatory of acetic acid (HAc), propionic acid (HPro), isobutyric acid (isoHBu), butyric acid (HBu), isovaleric acid (isoHVal), valeric acid (HVal) and caproic acid (HCa) concentrations in the CSTR and AnSBRs effluents measured as g COD L^−1^. TCOD_influent_ was the concentration of TCOD of the feedstock fed into the reactors (g COD L^−1^), SCOD_effluent_ was the soluble fraction of COD analysed in the semicontinuous reactors’ effluents (g COD L^−1^) and TCOD_effluent_ represented the TCOD concentration determined in the semicontinuous reactors’ effluents (g COD L^−1^).

## Results and discussion

### Temperature effect on VFAs production yields and profile

Beet molasses were firstly valorised via AF in batch mode to evaluate the effect of temperature on VFAs production yields and distribution profile. As can be seen in Table 2, similar VFAs concentration was reached at 25 °C and 35 °C, corresponding to bioconversion values of 62.0% and 61.1%, respectively. These high bioconversion efficiencies were mainly related to the high content of readily biodegradable carbohydrates present in beet molasses. Whereas remarkable low bioconversion efficiencies have been reported when feedstock with high content of proteins (33.9%)^49^ or complex carbohydrates (15%)^50^ were transformed into VFAs, similar values were found when carbohydrate-rich residues were studied under similar conditions (58.0%)^8^. These results revealed the suitability of the residue for VFAs production via AF.

**Table 2:** Composition of BCP test effluents achieved in the steady state of the process in terms of mean ± standard deviation (n = 3).

	BCP-25 °C		BCP-35 °C		BCP-55 °C	
pH	5.0	± 0.1	5.0	± 0.2	4.5	± 0.2
TCOD (g L^−1^)	37	± 1	43	± 1	44	± 2
SCOD/TCOD (%)	80.8	± 3.5	71.4	± 1.0	69.1	± 9.0
VFAs total (g L^−1^)	9.1	± 0.1	9.2	± 0.2	3.8	± 0.1
HAc (%)^a^	21.7	± 0.3	34.9	± 1.7	42.4	± 0.2
HPro (%)^a^	12.7	± 0.4	17.5	± 1.4	11.8	± 0.7
isoHBu (%)^a^	2.2	± 0.1	1.2	± 0.5	0.5	± 0.6
HBu (%)^a^	49.1	± 0.3	30.1	± 0.5	31.6	± 1.6
isoHVal (%)^a^	3.0	± 0.1	2.5	± 0.2	5.4	± 1.3
HVal (%)^a^	9.0	± 0.1	7.0	± 0.1	7.4	± 0.1
HCa (%)^a^	2.2	± 0.1	6.8	± 0.2	0.9	± 0.5
Lactic acid (g L^−1^)	< LD^b^		< LD^b^		5.4	± 0.1
Ethanol (g L^−1^)	< LD^b^		0.5	± 0.7	1.5	± 0.1
Bioconversion (%)	62.0	± 0.9	61.1	± 1.7	11.5	± 0.3
H_2_ yield (mL H_2_ g COD^−1^)	25.1	± 1.5	42.3	± 0.2	29.4	± 0.1
COD_acidified_ (%)	77.8	± 0.7	79.7	± 1.7	31.3	± 0.7

^a^VFA/TotalVFAs · 100.

^b^Lower than Limit of Detection.

In contrast, when the AF was operated at 55 °C, the bioconversion was only 11.5% (3.8 g VFAs L^−1^). The lower production of VFAs at 55 °C could be explained by an inhibition in the AF process. As it can be seen in Table 2, the solubilisation percentage (SCOD/TCOD) at 55 °C was very similar to those obtained at 35 °C (71.4% at 35 °C vs 69.1% at 55 °C), indicating that the hydrolysis step was not hampered. Indeed, the solubilisation percentage (SCOD/TCOD) determined for the three temperatures was high compared with previous studies performing batch experiments with carbohydrate rich feedstock^8^. By opposite, the acidification efficiency expressed as COD_acidified_ percentages was higher at lower temperatures than at 55 °C. (79.7% at 25 °C and 77.8% at 35 °C vs 31.3% at 55 °C). This fact indicated that the acidogenic step was drastically inhibited at 55 °C. In line with these results, Moretto et al.^51^ and He et al.^52^ found that even though thermophilic conditions favour organic matter solubilization (hydrolysis), the same did not occur for acidogenesis. It should be highlighted that the inoculum used for AF and the aforementioned studies were anaerobic mesophilic sludge collected from a conventional wastewater treatment plant. Since this inoculum was thriving at 35 ºC, the low acidification in BCP-55 °C could be related to a bacterial poor performance at temperature ranges other than 35 °C.

Beyond temperature, pH might have a more active role in acidogenesis inhibition^42^. The pH registered at the end of BCP-55 °C was 4.5, which was a low value for the acidogenic step and could explain the bioprocess failure. According to previous works, the optimum pH to produce VFAs is between 5 to 6, while lower pH leads to the accumulation of primary intermediates (lactic acid)^53,54^. The low pH value (< 5) and the low COD_acidified_ obtained at 55 °C suggested that the metabolic pathways that transform the primary metabolites into VFAs were hindered^30^. This fact was suppoted by the lactic acid (5.4 g L^−1^) accumulation in the BCP-55 °C (Table 2) and the low bioconversion of the substrate into VFAs. Although the AF at 55 °C was not successful for VFAs accumulation, it is worth noting that this inoculum worked properly at 25 °C and 35 °C, resulting in bioconversions higher than the ones reported in the literature for other carbohydrate-rich residues^8,12^.

Temperature also had an effect on VFAs distribution. Even though VFAs concentration was similar at 25 °C and 35 °C, the VFAs profiles were remarkably different. At both temperatures, HAc and HBu were the predominant VFAs. The prevalence of even-carbon VFAs was not surprising because this is a common feature of carbohydrate-rich substrates^55^. Nevertheless, their proportions varied from 21.7% of HAc at 25 °C to 34.9% at 35 °C and from 49.1% of HBu at 25 °C to 30.1% at 35 °C. Besides, one of the major differences was related to HCa concentration, being the highest concentration determined at 35 °C (6.8% vs 2.2% at 25 °C). BCP-35 °C also led to a higher H_2_ yield (42.3 mL H_2_·g COD^−1^) than BCP-25 °C (25.1 mL H_2_ g COD^−1^). The co-occurrence of HCa and H_2_ production has been previously observed when HCa production takes place via carbon chain elongation since this is a hydrogenogenic process^30^. These differences in the VFA profile could be related to changes in the microbial community due to differences in microbial growth rates depending on the temperature. The inocula used was an open-mixed culture that usually produce a mixed broth product^42^. Due to the existence of a great variety of microorganisms in the media, which provided a wide range of metabolic pathways, implementing different operational temperatures could lead to changes in the microbial community, and therefore in metabolic pathways. Some studies claim that an increase in temperature concominantly increases the metabolic rate. These differences are closely related to NADH + H + production, ending up as well in differing VFAs distribution profile^52^. Considering that similar VFAs production was reached at 25 °C and 35 °C, 25 °C was selected as the more convenient process temperature since the use of mild temperatures contributed to energy cost savings, allowing a scale-up of the process with lower operating costs. Moreover, as can be seen in Table 2, at 25 °C, C4–C6 chain VFAs production was enhanced (65%) when compared to 35 °C (47.6%). Taking into account that the VFA market value concomitantly increases with the carbon chain length^14^, 25 °C could be considered the most suitable temperature to maximize the cost-effectiveness of the process.

### Comparison of CSTR vs AnSBR performance: decoupling HRT and SRT

**Figure 2:**
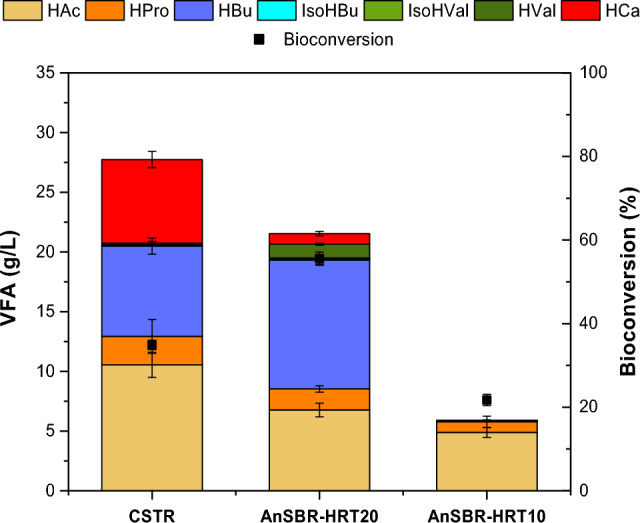
Effect of decoupling HRT and SRT, and effect of HRT on VFAs production, distribution and bioconversion efficiencies reached in the steady state of beet molasses AF.

Once 25 ºC was selected as the optimal process temperature to maximize VFAs, two different reactor configurations (CSTR and AnSBR) were evaluated to elucidate the effect of HRT and SRT on VFAs production in reactors operated in semicontinuous mode. As can be seen in Fig. 2 and Table 3, CSTR (HRT = SRT = 30 days) resulted in the highest VFAs concentration (27.6 g L^−1^), corresponding to a bioconversion efficiency of 34.9%.

**Table 3:** Composition of CSTR and AnSBR effluents determined in the steady state of the process in terms of average values ± standard deviation (n = 7).

	CSTR		AnSBR-HRT20^a^		AnSBR-HRT20^b^		AnSBR-HRT10^a^		AnSBR-HRT10^b^	
pH	5.8	± 0.2	5.9	± 0.2	5.9	± 0.2	5.8	± 0.1	5.8	± 0.1
TCOD (g L^−1^)	70.5	± 0.9	48.1	± 3.0	50.2	± 3.2	25.1	± 0.8	25.4	± 3.4
SCOD/TCOD (%)	95.9	± 2.6	96.7	± 5.8	95.1	± 6.3	95.2	± 2.0	94.3	± 3.5
TS (g L^−1^)	54.4	± 4.4	37.8	± 3.8	40.7	± 3.5	30.6	± 3.5	30.9	± 0.6
VS (g L^−1^)	34.4	± 2.6	22.8	± 3.1	24.8	± 2.6	18.0	± 0.9	18.9	± 0.8
VFAs total (g L^−1^)	27.6	± 1.7	21.5		± 0.6		5.9		± 0.4	
HAc (%)^c^	38.2	± 2.3	31.4		± 2.4		79.8		± 5.0	
HPro (%)^c^	8.4	± 4.9	8.1		± 1.2		18.8		± 5.4	
isoHBu (%)^c^	< LD^d^	< LD^d^	0.6		± 0.3		< LD^d^		< LD^d^	
HBu (%)^c^	27.7	± 2.1	50.0		± 1.1		1.2		± 0.3	
isoHVal (%)^c^	< LD^d^	< LD^d^	0.2		± 0.1		< LD^d^		< LD^d^	
HVal (%)^c^	3.1	± 0.7	5.4		± 0.4		< LD^d^		< LD^d^	
HCa (%)^c^	25.4	± 3.0	4.1		± 0.9		0.2		± 0.1	
Lactic acid (g L^−1^)	4.8	± 2.9	0.2		± 0.1		5.1		± 0.4	
Ethanol (g L^−1^)	0.5	± 0.6	< LD^d^		< LD^d^		0.1		< LD^d^	
Bioconversion (%)	34.9	± 1.1	55.5		± 1.5		21.7		± 1.3	
COD_acidified_ (%)	65.2	± 2.1	70.0		± 4.6		27.2		± 2.5	

^a^Supernatant phase.

^b^Sludge phase.

^c^VFAs/TotalVFAs ·100.

^d^Lower than Limit of Detection.

CSTR bioconversion efficiency was in accordance with previous studies performed with similar residues, such as the sugar industry wastewater (37% of bioconversion efficiency)^56^. Similarly, the acidification efficiency (COD_acidified_ = 65.2%) was also in the range of investigations dealing with carbohydrate-rich residues^12^, but lower when compared to agroindustrial wastes^8^ and cheese whey^23^. Given beet molasses exhibited an extremely high soluble COD (98.6% of the total COD, Table 1), it was expected that the organic matter was readily available for the microorganisms to ultimately produce VFAs. At this point, the significant concentration of lactic acid (4.8 g L^−1^) could be used as a chemical indicator of acidogenesis failure since the presence of this primary metabolite indicates that the secondary metabolites (VFAs) production pathways are not proceeding appropriately. Lactic acid and ethanol are primary intermediate metabolites that are normally easily converted into acetyl-CoA to form acetic acid, and also butyric acid though β-oxidation metabolic pathway^57^. Thus, lactic acid accumulation indicates that the metabolisms to produce VFAs were hindered.

To evaluate the HRT effect on acidogenesis performance, an alternative AnSBR configuration was used to decrease the HRT from 30 to 20 days while maintaining the SRT in 30 days. As it can be observed in Table 3, the reduction of HRT to 20 days resulted in a VFAs concentration decrease (from 27.5 to 21.5 g L^−1^). This fact was related to the dilution of the residue fed in the AnSBR. For both configuration (AnSBR and CSTR) to be comparable, reactors were fed with the same OLR. In this manner, HRT reduction involved an influent flow rate increase. A similar effect was also observed in the VFAs production from cheese whey valorization via AF^23^. Despite the lower concentration reached in the AnSBR in comparison to the CSTR, the bioconversion efficiency increased to 55.5%. The percentage of COD_acidified_ was slightly higher than the values attained for the CSTR, which could be explained by the low concentration of lactic acid measured in the AnSBR-HRT20 (0.2 g L^−1^). As a primary metabolite, the lower concentration of lactic acid in AnSBR than in CSTR suggested a more efficient conversion into VFAs. Thus, it could be concluded that lowering the HRT had a positive effect on acidogenesis stage, opening the possibility to treat larger amounts of organic matter while obtaining better bioconversion efficiencies.

It is also important to highlight another relevant advantage of AnSBR configuration when targeting at VFAs production. This configuration allowed obtaining a supernatant with a lower content of solids (TS = 37.8 g L^−1^) than those resulting from the CSTR configuration (TS = 54.4 g L^−1^). The separation of streams in the AnSBR-HRT20 configuration could facilitate the recovery of VFAs by reducing the complexity and the cost of downstream processes. Likewise, it should be stressed that the proper performance of reactors does not only rely on the population abundance but also their activity. In this way, AnSBR-HRT20, exhibiting a lower VS content, provided better results in VFAs yields than the CSTR. Based on the results obtained herein, lowering the HRT mediated the development of a specialized microbiome with proper activities for VFAs production.

VFAs profile was also strongly affected by both reactor configuration and decoupling of SRT and HRT. As observed in BCP tests, the main differences were registered for even-chain VFAs. The VFA pool produced in AnSBR-HRT20 was dominated by HBu (50.0%) followed by HAc (31.4%). By contrast, in the CSTR, HBu abundance was 22 percentual points lower than in AnSBR-HRT20 whereas HCa was 21 percentual points higher than in AnSBR-HRT20. The increased HCa values obtained in the CSTR were in agreement with previous investigations dealing with the AF of carbohydrate-rich residues^12^. As mentioned above, HCa is mainly produced via carbon chain elongation when mildly acidic pH values are set^54^. This fact can be explained based on the longer HRT of the CSTR, which allowed the growth of specialized microorganisms responsible for chain elongation and H_2_ production, including the primary metabolites producers^12,58^, such as *Ruminococcus*^12,46^ and members of *Lachnospiraceae* family^46^ identified in previous works related to VFAs production from carbohydrate-rich feedstocks. Thus, the lower HRT implemented in the AnSBR seems to reduce the primary metabolites accumulation, which acts as essential electron donors in the elongation process. This fact could be the main reason for the reduced content of HCa in AnSBR since the elongation was limited.

In summary, the decrease in HRT had a positive effect on VFAs production efficiency, opening the possibility to treat larger amounts of organic matter while obtaining better bioconversions to VFAs and keeping an optimal microbial activity. This increase in bioconversion efficiency entailed a remarkable decrease in HCa, which is important to be considered depending on the VFAs application.

### Effect of HRT in AnSBR when targeting at VFA production

Given the enhanced bioconversion efficiency attained by decoupling the SRT and HRT in the AnSBR configuration, a further decrease in the HRT to 10 days was evaluated in a second AnSBR (AnSBR-HRT10) while maintaining the SRT at 30 days. This decrease would imply an increase in volumetric flows to be fed in the reactors and thus, a faster bioconversion could be achieved.

As it can be seen in Table 3, the VFA production in AnSBR-HRT10 decreased to 5.9 g L^−1^, notably low when compared to the production achieved at an HRT of 20 days. As it occurred with the difference between the CSTR and the AnSBR-HRT20, this might be due to the increased flow required for reaching this HRT while maintaining the same OLR for both AnSBR (3 g COD L^−1^ day^−1^). Nevertheless, in this case, the bioconversion efficiency also strongly decreased from 55.5% (AnSBR-HRT20) to 21.7% (AnSBR-HRT10). Accordingly, COD_acidified_ was also very low (27.2%) compared to AnSBR-HRT20, indicating an inhibition of the acidogenic step as the substrate was not converted into VFAs, but lactic acid was accumulating in the system (5.1 g L^−1^). The reduction of HRT to 10 days also affected the VFAs distribution. HAc was the main VFA produced in AnSBR-HRT10, accounting for 79.8% of the total VFAs (Table 3). The production of HPro was also higher in AnSBR-HRT10 (18.8%) than in AnSBR-HRT20 and in the CSTR. However, long-chain VFAs (C_4_ carbon chain and longer) were barely produced. This change in the products profile confirmed the inhibition of the acidogenic step, suggesting that HRT varied the metabolic pathways occurrence. Whereas 30 days of HRT in CSTR corresponded to carbon chain elongation that resulted in HCa production, 20 days of HRT in AnSBR-HRT20 promoted a butyrate-type fermentation (high HBu accumulation). These metabolic pathways were inhibited in AnSBR-HRT10, as denoted in the accumulation of acetic acid and lactic acid, being lactate-type fermentation the main metabolic pathway^40^. In this case, it seemed that the sludge of AnSBR-HRT10 was a lactic acid specialised producing microbiome, such as *Lactobacillus*^30^* or Lachnospiraceae*^12^ that have been previously identified when lactic acid prevailed over VFAs.

In this case, the further decrease of the HRT to 10 days was not beneficial for VFAs production since AF efficiency decreased and the product distribution had changed significantly. Nevertheless, the production of lactic acid was improved, proving that those conditions (lower HRT and higher flow rates) could direct the AF process to the production of other high-added value products interesting for the industry, such as lactic acid that is a precursor of bioplastics.

## Conclusions

Aiming at substituting petroderivatives by renewable biochemicals, this investigation showed that beet molasses is a suitable feedstock to efficiently produce carboxylates (VFAs) via anaerobic fermentation (AF). At an operating temperature of 25 °C the overall production of VFA and, in particular, long-chain VFAs (C4–C6) yields were maximized, allowing a future cost-effectiveness scale up of the process given the low energy requirement. In addition, this research has proven the benefits of decoupling the HRT and the SRT by changing reactor configurations (from CSTR to AnSBR). Thus, the manipulation of HRT by using an AnSBR improved AF efficiency by increasing VFAs production yield. By contrast, in a CSTR, the enhancement of biological carbon chain elongation was achieved at HRT of 30 days. Long HRTs allowed the microorganisms to produce and accumulate more HCa in the medium, but at the expense of a lower process efficiency than in AnSBR-HRT20. Lowering the HRT from 20 to 10 days in the AnSBR configuration had a negative effect on bioconversion efficiency. Moreover, as a consequence of the increasing flow rates needed to implement a lower HRT, VFAs profile completely changed due to a shift from the butyrate-type fermentation to a lactate-type fermentation.

## Data Availability

The datasets used and/or analysed during the current study are available from the corresponding author upon reasonable request. Other data generated or analysed during this study are included in this article.
